# Two new species of the genus *Timia* and a redescription of *Timia mongolica* (Diptera, Ulidiidae)

**DOI:** 10.3897/zookeys.615.9311

**Published:** 2016-09-07

**Authors:** Tatiana V. Galinskaya

**Affiliations:** 1 Entomology Department, Biological Faculty, Lomonosov Moscow State University, Moscow, 119234 Russia

**Keywords:** Diptera, new species, redescription, Timia, Ulidiidae

## Abstract

Two new species of the genus *Timia* Wiedemann, 1824 are described and illustrated. *Timia lazebnayae* **sp. n.** from Uzbekistan has yellow body and is similar to *Timia gobica* Zaitzev, 1982, differing from it only by the entirely yellow flagellomere 1. *Timia shatalkini* **sp. n.** from Mongolia has dark body and differs from all other dark-colored representatives of the genus by the cell r_4+5_ being completely closed, forming petiole at the wing apex. *Timia mongolica* Zaitsev, 1982 is redescribed and an updated key for yellow-coloured *Timia* is provided.

## Introduction

*Timia* Wiedemann, 1824 is a Palaearctic genus which includes 60 described species, commonly found in semi-arid and arid regions (Becker 1906; Hendel 1908; Hennig 1940; Zaitzev 1982; Zaitzev 1984a, b; Kameneva 1996, 2000, 2010; Galinskaya 2011, 2014; Morgulis and Freidberg 2014). While studying the Ulidiidae material from the collection of the Zoological Institute of the Russian Academy of Sciences, Saint-Petersburg (ZISP), the author came across several specimens of flies, belonging to the two new species described below. Examination of the type material of *Timia mongolica* Zaitsev, 1982 revealed several disparities between the specimens and the description of the species (Zaitsev 1982); for example, the body appeared to be yellow and not dark brown. The previously unknown female of *Timia mongolica* was found in the collection of ZISP. Therefore, this species is redescribed here and an updated key for the yellow-coloured *Timia* is provided.

## Material and methods

Specimens examined were obtained from or deposited in the collections of the following institutions:

ZISP
 Zoological Institute of the Russian Academy of Sciences, Saint-Petersburg, Russia 


ZMUM
Zoological Museum of the Lomonosov State University, Moscow, Russia 


MNKB
Museum für Naturkunde, Leibniz-Institut für Evolutions und Biodiversitätsforschung, Berlin, Germany 


Morphological terminology and abbreviations generally follow White et al. (1999). Series of photos were taken directly by the Canon EOS 40D camera with Canon MP-E 65 mm lens and then composed with CombineZM software (Hadley 2007). Measurements are provided in millimetres.

## Taxonomy

### Timia

Taxon classificationAnimaliaDipteraUlidiidae

Wiedemann 1824

#### Type species.

*Timia erythrocephala* Pallas in Wiedemann, 1824 (by monotypy).

#### Diagnosis.

Yellow or black flies. Frons usually with dents and bumps, shiny or almost shiny, sometimes with white microtrichose areas. Antennal grooves deep, oval, well-separated by wide facial carina. Thorax and abdomen shining or shagreened, sometimes almost matt, sometimes with green metallic shine, often with microtrichose areas. Wing hyaline, in some species with dark cells bc, c, sc and apical spot. Male genitalia: epandrium subovoid; surstylus hook-like, sometimes with marked postero-dorsal lobe; cerci clearly bilobed; phallus long, coiled and partially flattened divided into two parts, with a pair of sclerotized taeniae ending approximately at its mid-length and another pair of taeniae beginning at phallus middle almost reaching phallus apex; phallus apical half bearing long membranous appendix (“caecum”); distiphallus apex bowed and bearing numerous spurs, and “glans” formed by hooks or lobes surrounding gonopore. Surstylus hook-like, sometimes with marked postero-dorsal lobe. Cerci clearly bilobed. Female terminalia: aculeus moderately long and wide, with short oval cercal unit; three spherical spermathecae with smooth or micropapillose surface. Separation of *Timia* and *Ulidia* Meigen, 1826 is difficult. The characters used so far are mainly as follow: frons smooth (in *Timia*) or dimpled (in *Ulidia*) (with some exceptions), head and thorax microtrichose (in *Timia*) or bare (in *Ulidia*, but some species assigned to *Timia* have shiny head and thorax, and *Ulidia metope* Kameneva, 2010 has frons widely microtrichose) (Chen and Kameneva 2009; Kameneva 2010). In addition, species of the genus *Ulidia* always have entirely black bodies without yellow parts of thorax and abdomen.

#### Remarks.

Adult *Timia*, as well as many other ulidiids, are attracted to various organic residues (decaying plant tissue, animal corpses, excrements). In arid habitats, the surface of organic residues is quickly covered with a dried crust, under which semi-liquid substrate is preserved for a relatively long time. The proboscis of *Timia* has longitudinal rows of pointed outgrowths located on the labellum; flies make a hole in a crust with these appendages and then penetrate with long tubular appendage of the proboscis into it, absorbing semi-liquid substrate (Zaitzev 1982).

### Key to the yellow-bodied species of the genus *Timia* and *Timia shatalkini* sp. n.

**Table:** 

1	Body yellow (Figure 1)	**2**
–	Body black (Figure 2)	**11**
2	Parafacialium wider than flagellomere 1 and twice as wide as antennal groove (Figure 10)	**3**
–	Parafacialium as wide as or narrower than flagellomere 1, and narrower than antennal groove (Figure 1)	**4**
3	Posteroapical extension of cell bcu more than 1.5 times as long as maximum width of cell. Mesonotum shining, without microtrichose areas (Figure 10). Female: cercal unit with 1 pair of long basal setae. Male: cerci rounded apically	***Timia testacea* Portschinsky, 1891**
–	Posteroapical extension of cell bcu at most as long as cell width at its middle. Mesonotum silvery white microtrichose, with rows of merging shining spots around setae (Figure 11). Female: cercal unit with 2 pairs of long setae. Male: cerci with obtuse angulate apex	***Timia zaitzevi* Galinskaya, 2011**
4	Cell r_4+5_ open. Parafacialium almost as wide as flagellomere 1 (Figure 6)	**6**
–	Cell r_4+5_ completely closed, forming petiole at wing tip. Parafacialium less than half as wide as flagellomere 1 (Figure 8)	**5**
5	Flagellomere 1 entirely black (Figure 8)	***Timia gobica* Zaitzev, 1982**
–	Flagellomere 1 entirely orange (Figure 1)	***Timia lazebnayae* sp. n.**
6	Distance between apices of veins R_4+5_ and M longer than cross-vein R-M. Scape and pedicel entirely black, contrasting with yellow flagellomere 1 (Figure 6)	***Timia dimidiata* Becker, 1906**
–	Distance between apices of veins R_4+5_ and M shorter than cross-vein R-M. Scape and pedicel entirely yellow (or brown-yellow, in this case flagellomere 1 black)	**7**
7	Flagellomere 1 and apex of palpus yellow. Distance between apices of veins R_4+5_ and M more than half as long as cross-vein R-M. Male fore femur with moderately long and thin setae (Figures 4, 7, 9)	**9**
–	Flagellomere 1 and palpus apex black or dark brown. Distance between apices of veins R_4+5_ and M 0.1 as long as cross-vein R-M. Male fore femur either with spines or long and thin setae (Figures 3, 5)	**8**
8	Frons yellow, with black triangle medially, two times wider and six times longer than ocellar triangle. Thorax with a black pattern. Occiput black medially, narrow yellow laterally (Figure 3). Male fore femur with posteroventral series of short black spines	***Timia mongolica* Zaitsev, 1982**
–	Frons yellow, with black ocellar triangle. Thorax with a light brown pattern. Occiput yellow. Male fore femur with thin setulae, without posteroventral series of short black spines (Figure 5)	***Timia canaliculata* Becker, 1906**
9	Microtrichose areas on mesonotum forming a medial vitta and spots of various shapes at its sides (Figures 4, 9)	**10**
–	Mesonotum entirely silvery white microtrichose, without rows of merging shining spots around setae (Figure 7)	***Timia flaveola* Galinskaya, 2011**
10	Mesonotum with lateral vittae of silver microtrichia extending posterior to wing level. Parafacialium >0.25 times as wide as eye. Posteroapical extension of cell bcu shorter than (0.75 times as long as) width of the cell at its middle. Face >1.4 times wider than high (1.85 times as wide as its height) (Figure 4)	***Timia berlandi* Séguy, 1953**
–	Lateral vittae of silver microtrichia not extending posterior to wing level. Parafacialium <0.25 times as wide as eye. Posteroapical extension of cell bcu longer than (1.7 times as long as) width of the cell at its middle. Face <1.4 times wider than high (as wide as high) (Figure 9)	***Timia orientalis* Zaitzev, 1982**
11	Cell r_4+5_ apically completely closed, forming petiole. Parafacialium about half as wide as flagellomere 1. Thorax with microtrichose area on mesonotum forming a medial vitta; thorax light brown, with dark brown lateral spots (mainly on presutural area of scutum) and medial stripes (Figure 2)	***Timia shatalkini* sp. n.**
–	Cell r_4+5_ open. Parafacialium width variable. Thorax with or without pattern of microtrichia, but without a medial vitta; thorax coloration variable, predominantly uniformly black or black with yellow scutellum	**other *Timia* species**

**Figure 1.**
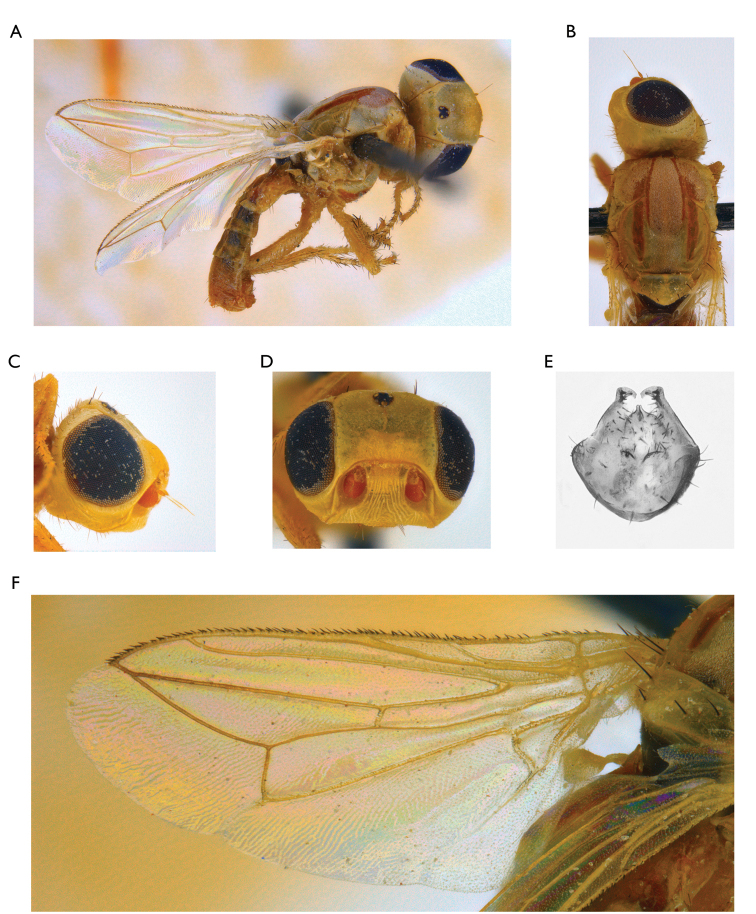
*Timia lazebnayae* Galinskaya sp. n., paratype male; **A** habitus, lateral view **B** head and thorax, dorsal view **C** head, lateral view **D** head, anterior view **E** epandrium, dorsal view **F** wing.

**Figure 2.**
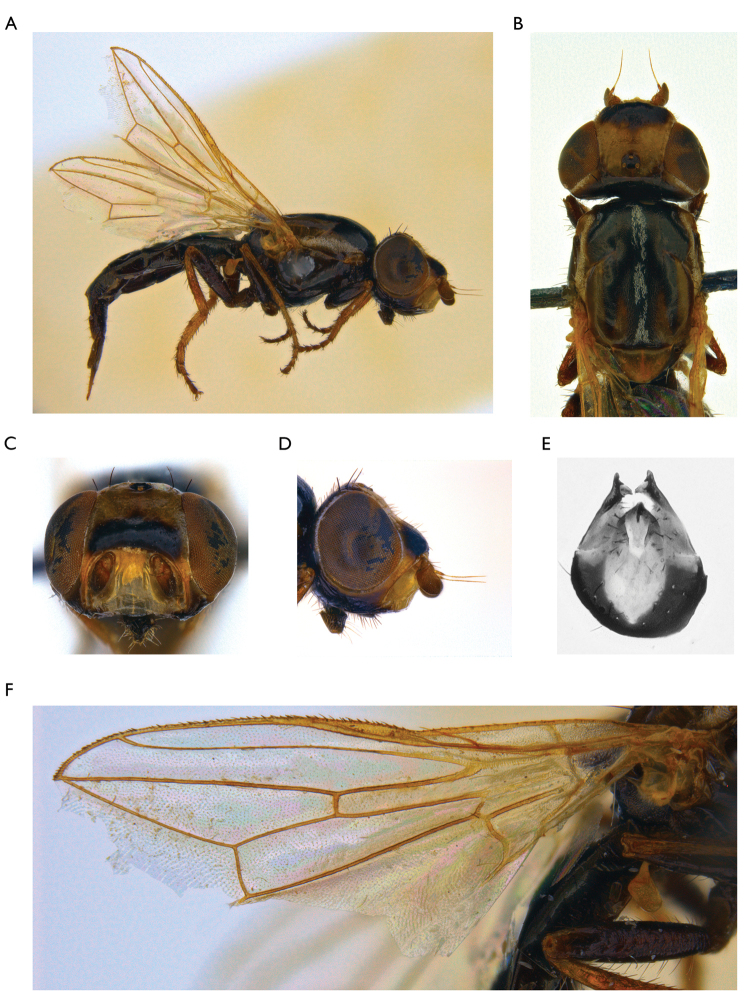
*Timia shatalkini* Galinskaya, sp. n., paratype female; **A** habitus, lateral view **B** head and thorax, dorsal view **C** head, anterior view **D** head, lateral view **E** epandrium of male, dorsal view **F** wing.

**Figure 3.**
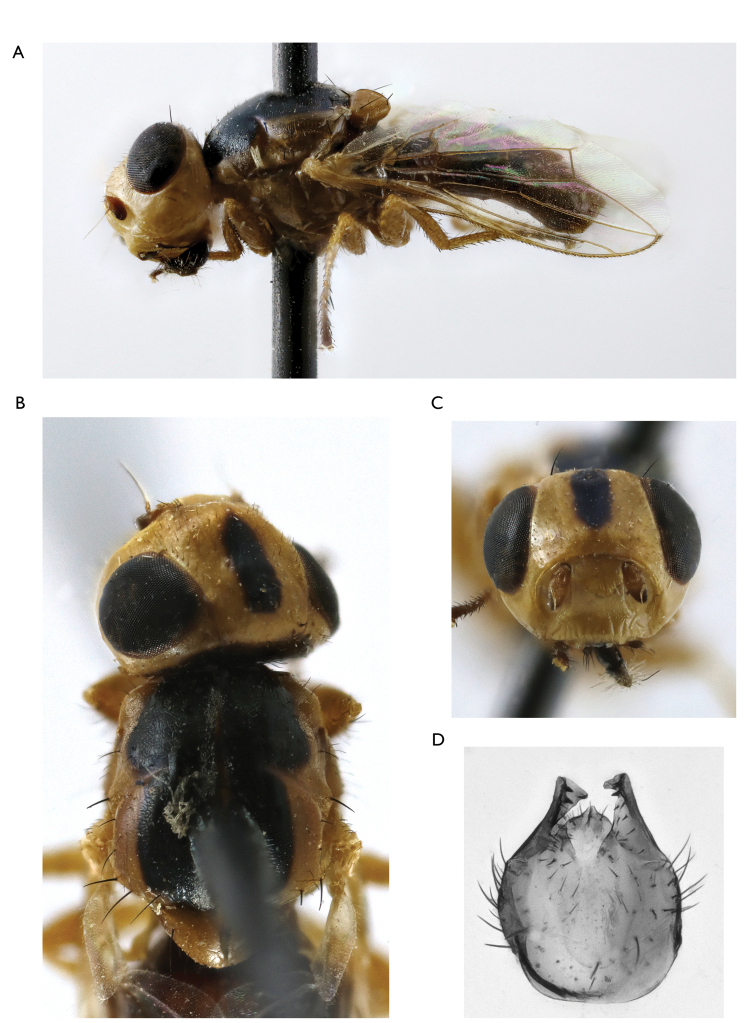
*Timia mongolica* Zaitzev, 1982, paratype male **A** habitus, lateral view **B** head and thorax, dorsal view **C** head, anterior view **D** epandrium, dorsal view.

**Figure 4.**
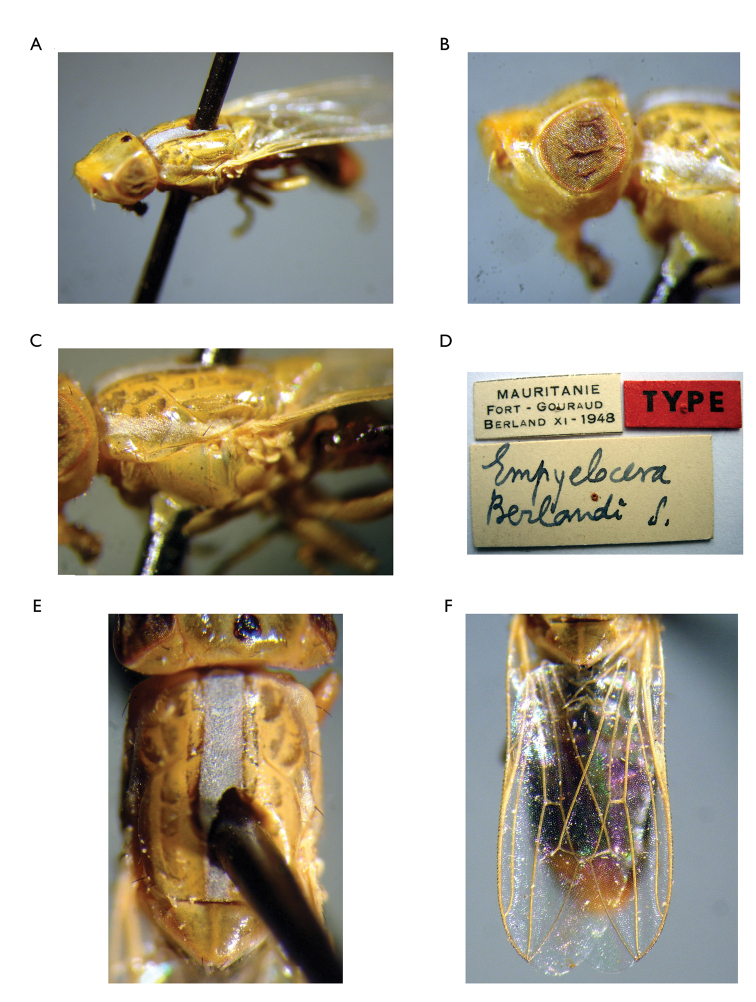
*Timia berlandi* (Seguy, 1953), holotype male **A** habitus, lateral view **B** head, lateral view **C** thorax, lateral view **D** label **E** thorax, dorsal view **F** wing (after Kameneva 2010, with permission).

**Figure 5.**
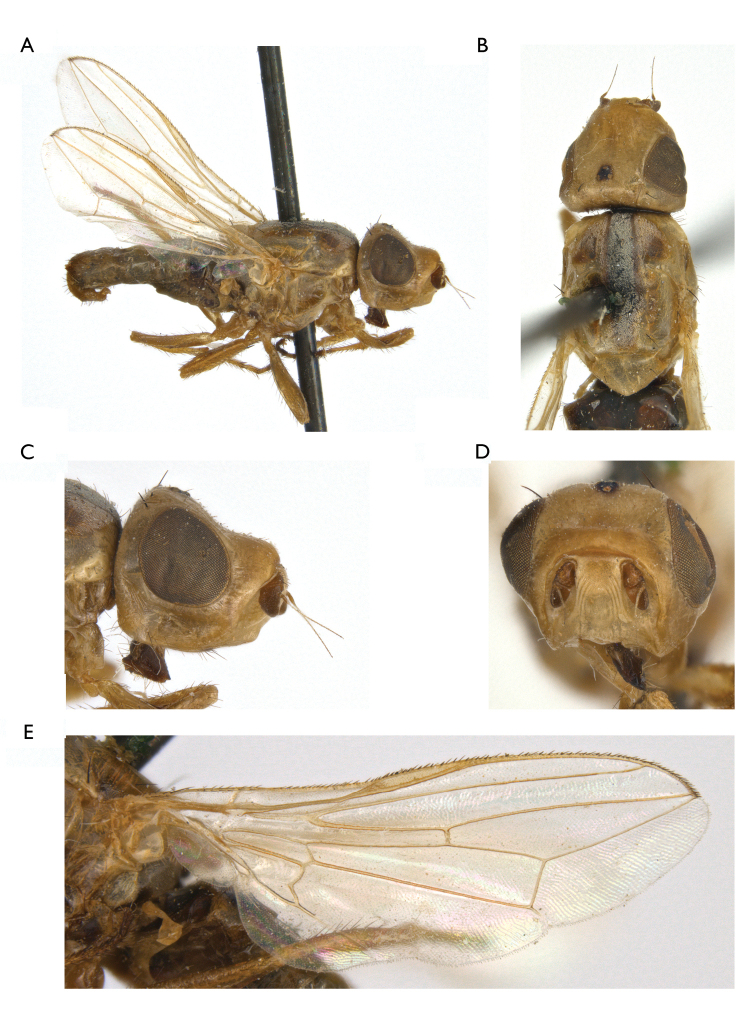
*Timia canaliculata* Becker, 1906, lectotype male **A** habitus, lateral view **B** head and thorax, dorsal view **C** head, lateral view **D** head, anterior view **E** wing.

**Figure 6.**
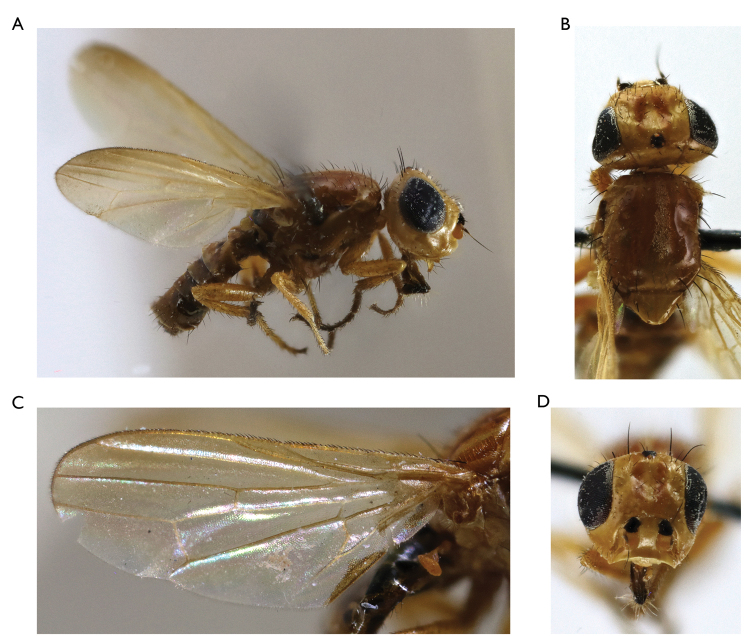
*Timia dimidiata* Becker, 1906, male **A** habitus, lateral view **B** head and thorax, dorsal view **C** wing **D** head, anterior view.

**Figure 7.**
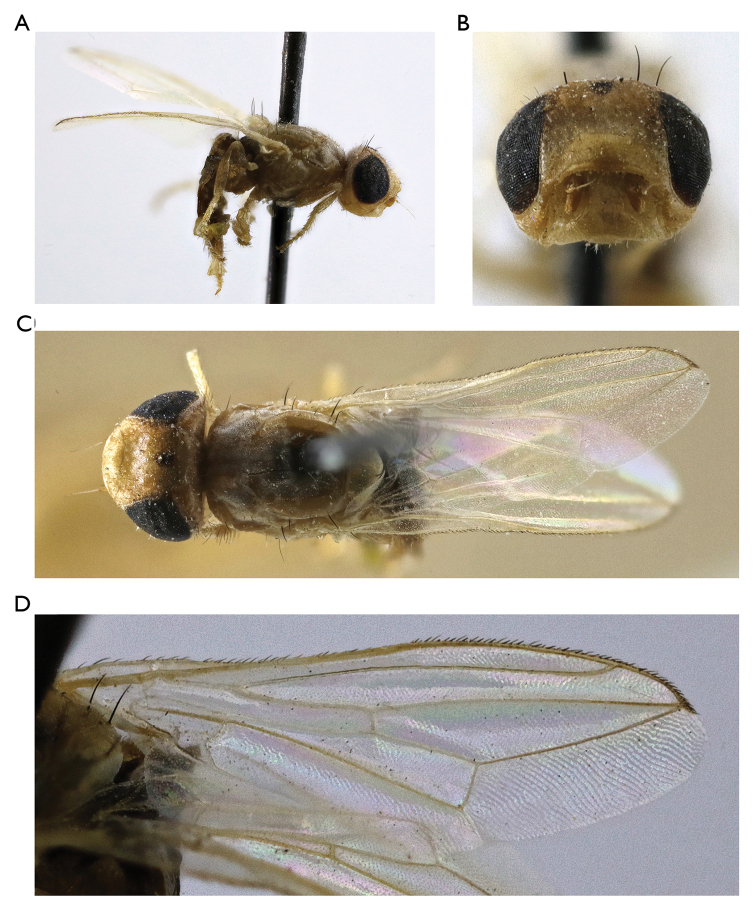
*Timia flaveola* Galinskaya, 2011, paratype male **A** habitus, lateral view **B** head, anterior view **C** head and thorax, dorsal view **D** wing.

**Figure 8.**
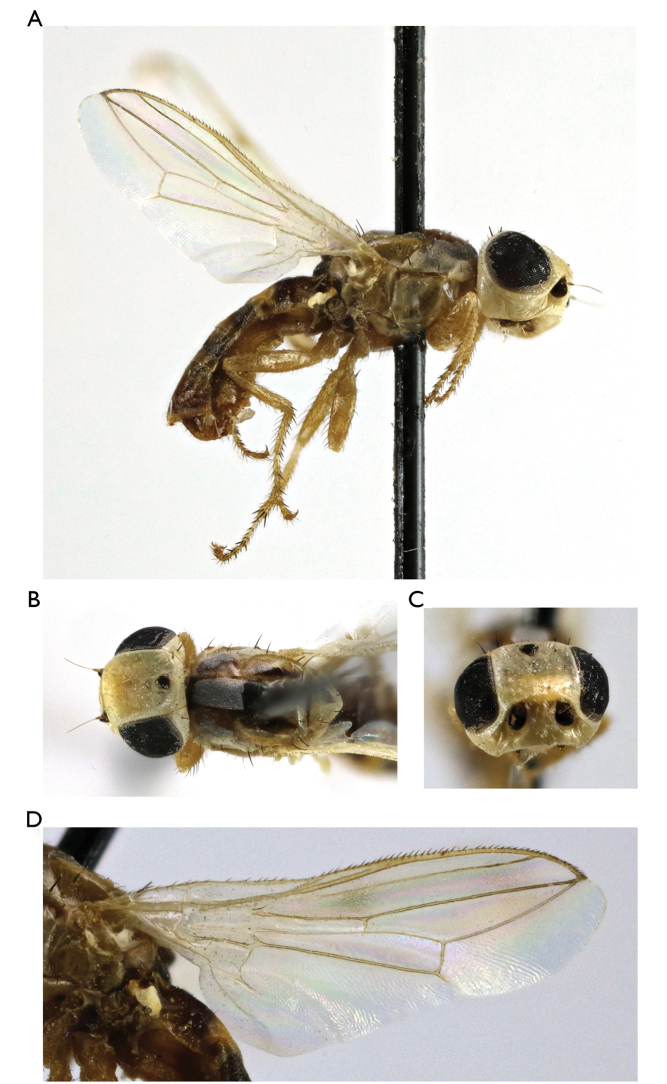
*Timia gobica* Zaitzev, 1982, male **A** habitus, lateral view **B** head and thorax, dorsal view **C** head, anterior view **D** wing.

**Figure 9.**
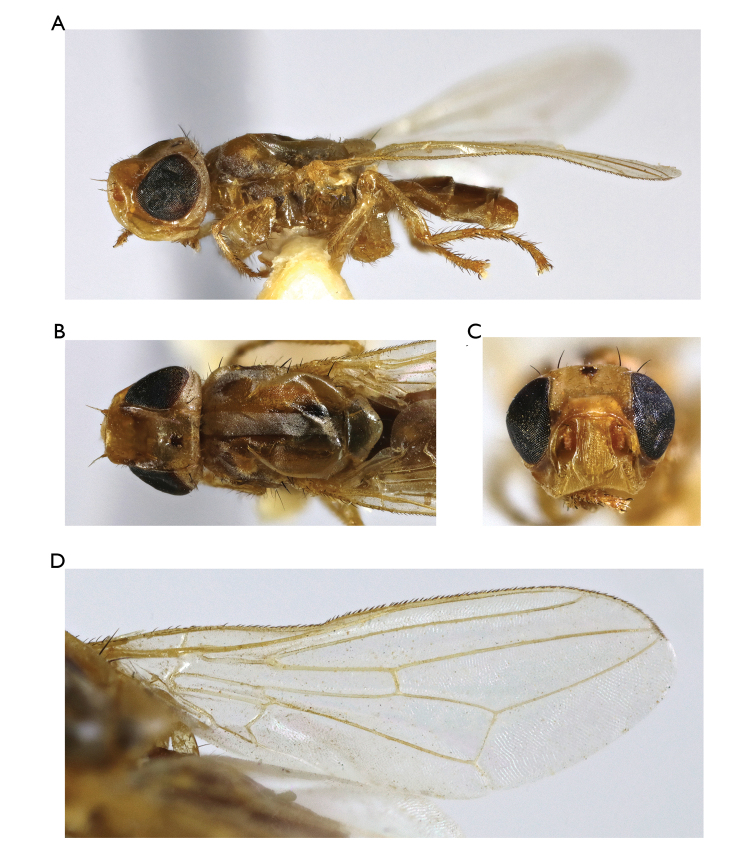
*Timia orientalis* Zaitzev, 1982, male **A** habitus, lateral view **B** head and thorax, dorsal view **C** head, anterior view **D** wing.

**Figure 10.**
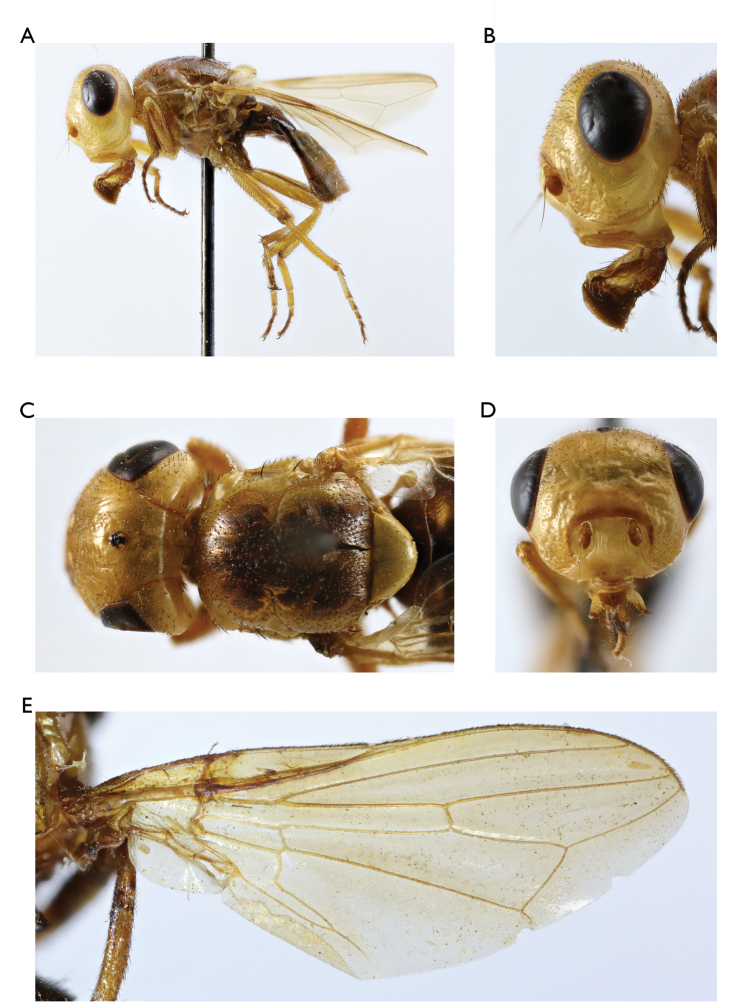
*Timia testacea Portschinsky*, 1891, male **A** habitus, lateral view **B** head, lateral view **C** head and thorax, dorsal view **D** head, anterior view **E** wing.

**Figure 11.**
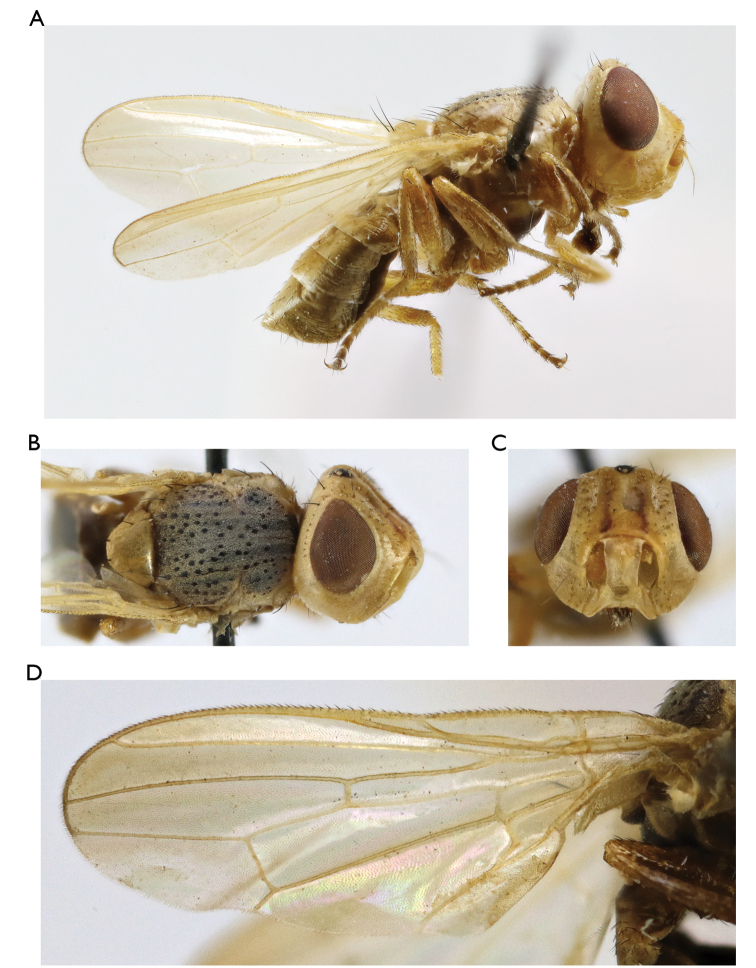
*Timia zaitzevi* Galinskaya, 2011, paratype male **A** habitus, lateral view **B** head and thorax, dorsal view **C** head, anterior view **D** wing.

### Timia lazebnayae sp. n.

Taxon classificationAnimaliaDipteraUlidiidae

http://zoobank.org/498E6DD0-14BF-4AD3-B0FD-17A2C59B0E4B

Figure 1

#### Type material.

***Holotype male*: Uzbekistan**: “70 км СЗ Газли, пески Кызыл Кум, Зайцев, 27.V.1965” [70 km NW Gazli, sands of Kyzyl Kum, Zaitzev leg. 27.V.1965] (ZISP).

***Paratype*: Uzbekistan**: 1 male, same data as holotype (ZISP).

#### Diagnosis.

*Timia lazebnayae* sp. n., *Timia shatalkini* sp. n. and *Timia gobica* differ from all other *Timia* species by cell r_4+5_ completely closed, forming petiole at wing tip and by parafacialium narrow, almost 0.3 times as narrow as first flagellomere. *Timia lazebnayae* sp. n. differing from *Timia shatalkini* sp. n. and *Timia gobica* by first flagellomere entirely yellow. *Timia lazebnayae* sp. n. is characterised by body yellow; cell r_4+5_ apically completely closed, forming petiole at wing tip; parafacialium less than half as wide as flagellomere 1, and narrower than antennal groove; flagellomere 1 entirely orange.

#### Description.

**Male**: *Head*. Frons pale yellow, evenly covered with short and thin brown setulae. Frons with band of weak microtrichosity along eye margin. Ocellar triangle shining black. Lateral part of vertex entirely yellow without dark markings. Occiput pale yellow, covered with short and thin black setulae. Medial occipital sclerite yellow; lateral occipital sclerite yellow, with silver-white microtrichosity. Gena yellow, 0.25 times as low as eye. Postgena yellow. Parafacialium yellow, 0.3 times as narrow as antennal grooves, 0.5 times as narrow as flagellomere 1. Eye as high as wide. Lunula pale yellow. Face yellow, without darkened band along the ventral edge. Clypeus yellow. Scape and pedicel yellow. Flagellomere 1 completely yellow, matted, roundish. Arista yellow. Antennal groove yellow, with thin silver-white microtrichosity. Proboscis yellow, slightly darkened distally. Palpus yellow.

*Thorax*. Yellow, with pale brown lateral spots (mainly on presutural area of scutum) and with medial stripe. Mesonotum with silver-white microtrichosity, lateral spots and medial stripe. Postpronotum slightly microtrichose. Scutellum nearly shiny, yellow, with short black setulae ventrally. Mediotergite dark brown. Pleura yellow, slightly microtrichose on dorsal edge of anepisternum. Pleura covered with sparse short black setulae. Katepisternum with pale brown spot in ventral portion.

*Setae*. Two small orbital setae (dorsal seta little longer than ventral seta), one divergent ocellar seta, one divergent postocellar seta, one long divergent lateral vertical seta, five divergent postocular setae, one short paravertical seta, and one long convergent medial vertical seta. Three postgenal setulae, one long genal seta. Two groups of supracervical setae over occipital foramen. One postpronotal seta, two notopleural setae, one supra-alar seta, one prescutellar dorsocentral seta, one postalar seta, one intra-alar seta; two scutellar; anepimeral setae absent; one anepisternal seta, one katepisternal seta, anepisternum and katepisternum covered with short black setulae.

*Legs*. Entirely yellow.

*Wings*. Hyaline. Cell r_4+5_ completely closed, forming petiolate at wing apex. Posteroapical extension of cell bcu 0.2 times as short as width of the cell at its middle. Halter yellow.

*Abdomen*. Yellow, non-microtrichose.

*Male genitalia*. Surstylus with anteroventral lobe, curved proximally at right angle, bearing two spines at its medial margin; and with short posteromedial lobe covered with setulae ventrally. Cerci tapered at apex, separated by narrow slit, covered with long setulae.

Body length, 3.5–3.6 mm. Wing length, 3.1–3.2 mm.

**Female**: unknown.

#### Etymology.

The name is in dedication to a good friend V.S. Lazebnaya.

#### Distribution.

Uzbekistan.

### Timia shatalkini sp. n.

Taxon classificationAnimaliaDipteraUlidiidae

http://zoobank.org/FC9FEE83-97B7-4CBC-8130-F7DF09D5F2E0

Figure 2

#### Type material.

***Holotype male*: Mongolia**: “Монголия, Кобдоский аймак, Ур. Елхон, 20 км ЮВ Алтая на Бодончи, Кержнер, 27.VII.1970” [Khovd Province, Elhon, 20 km SE Altai to Bodonchi, 27.VII.1970, I.M. Kerzhner leg.] (ZISP).

***Paratype*: Mongolia**: 1 female, label as in the holotype .

#### Diagnosis.

*Timia shatalkini* sp. n., *Timia lazebnayae* sp. n. and *Timia gobica* differ from all other *Timia* species by cell r_4+5_ completely closed, forming petiole at wing tip and by parafacialium narrow, almost 0.3 times as narrow as flagellomere. *Timia shatalkini* sp. n. differing from *Timia lazebnayae* sp. n. and *Timia gobica* by dark brown body. *Timia shatalkini* sp. n. is characterised by body black; cell r_4+5_ apically completely closed, forming petiole at wing tip; parafacialium about half as narrow as flagellomere 1. Thorax with microtrichose area on mesonotum forming a medial vitta; thorax light brown, with dark brown lateral spots (mainly on presutural area of scutum) and medial stripes.

#### Description.

**Male**: *Head*. Frons pale brown dorsally, dark brown ventrally, evenly covered with short and thin brown setulae. Frons with band of weak microtrichosity along eye margin. Ocellar triangle shining black. Lateral part of vertex brown. Occiput brown, covered with short and thin black setulae. Medial occipital sclerite brown; lateral occipital sclerite brown, with band of weak microtrichosity along eye margin. Gena 0.25 times as low as eye, brown, with yellow band along eye margin. Postgena brown. Parafacialium yellow, 0.3 times as narrow as antennal groove, 0.5 times as narrow as flagellomere 1. Eye as high as wide. Lunula pale yellow. Face yellow, with darkened band along the ventral edge. Clypeus yellow. Scape and pedicel light brown. Flagellomere 1 light brown proximally, dark brown distally, matt, roundish. Arista light brown. Antennal groove yellow, slightly darkened. Proboscis dark brown. Palpus dark brown.

*Thorax*. Pale brown, with dark brown lateral spots (mainly on presutural area of scutum) and medial stripe. Mesonotum with silver-white microtrichose medial stripe. Postpronotum with silver-white microtrichosity. Scutellum subshining light brown, with short black setulae over dorsal surface. Mediotergite dark brown. Pleura dark brown, slightly microtrichose on dorsal edge of anepisternum. Pleura covered with sparse short black setulae.

*Setae*. Four small orbital setae (dorsal orbital seta little longer than other ones), one divergent ocellar seta, one divergent postocellar seta, one long convergent medial vertical seta, five divergent postocular setae, one short paravertical seta, and one long divergent lateral vertical seta. Three genal setulae, five postgenal setulae, one long genal seta. Two groups of supracervical setae over occipital foramen.

One postpronotal seta, two notopleural setae, one supra-alar seta, one prescutellar dorsocentral seta, one postalar seta, one intra-alar seta; two scutellar; anepimeral setae absent; one anepisternal, one katepisternal seta, also anepisternum and katepisternum covered with short black setulae.

*Legs*. Coxa, trochanter and femur dark brown, tibia and tarsus yellow.

*Wings*. Hyaline. Cell r_4+5_ completely closed, forming petiolate at wing apex. Posteroapical extension of cell bcu 0.4 times as long as the width of cell at its middle. Halter yellow.

*Abdomen*. Dark brown, non-microtrichose.

*Male genitalia*. Surstylus with anteroventral lobe curved proximally at right angle, bears two spines at its medial margin; and with short posteromedial lobe covered with setulae on its ventral side. Cerci tapered apically, separated by narrow slit, covered with long setulae.

Body length 3.1 mm. Wing length 2.3 mm.

**Female**: similar to male, except genital structures.

Body length 5.1 mm. Wing length 3.2 mm.

#### Etymology.

Named in honor of Dr. A.I. Shatalkin, supervisor of my PhD thesis.

#### Distribution.

Mongolia.

### Timia mongolica

Taxon classificationAnimaliaDipteraUlidiidae

Zaitzev, 1982

Figure 3

#### Type material.

***Holotype male*: Mongolia**: “Монголия, Баян-Хонгорский аймак, 30 км ССВ родн. Шара-Хулсны-Булак, Зайцев, 4.IX.1970” Bayankhongor Province, 30 km NNE spring Shara Hulsny-Bulak, 4.IX.1970, Zaitzev leg. (ZISP).

***Paratype*: Mongolia**: 1 male, label as in the holotype (ZISP).

***Additional material*: Mongolia**: 3 males, 1 female, label as in the holotype (ZISP).

#### Redescription.

**Male**: *Head*. Frons yellow, with large black triangle medially, without microtrichosity, evenly covered with short and thin black setulae. Ocellar triangle shining black. Lateral part of vertex entirely yellow without dark marks. Occiput yellow, medially black, slightly microtrichose close to eye margin, covered with short and thin black setulae. Gena yellow, 0.4 times as low as eye. Postgena yellow. Parafacialium yellow, 0.5 times as narrow as antennal grooves and as wide as flagellomere 1. Eye 1.3 times as high as wide. Lunula yellow. Face yellow, without narrow darkened band along the ventral edge. Clypeus pale yellow, darkened along ventral edge. Scape and pedicel light brown. Flagellomere 1 brown at base, almost black at apex, matted. Arista light brown. Antennal groove yellow, with thin silver-white microtrichosity. Proboscis brown. Palpus brown at base, black at apex.

*Thorax*. Yellow, with black pattern. Mesonotum with silver-white microtrichose medial stripe. Postpronotum with silver-white microtrichosity. Scutellum subshining yellow, faintly sculptured, with short black setulae over its ventral surface. Mediotergite yellow, with brown spot at the middle. Pleura yellow.

*Setae*. Four small orbital setae (dorsal orbital seta little longer than other ones), one divergent ocellar seta, one divergent postocellar seta, one long divergent lateral vertical seta, four divergent postocular setae, one short paravertical seta and one long convergent lateral vertical seta. Four genal setulae, eight postgenal setulae, one long genal seta. Two groups of supracervical setae over occipital foramen.

One postpronotal seta, two notopleural setae, one supra-alar seta, one prescutellar dorsocentral seta, one postalar seta, one intra-alar setae, two scutellar; anepimeral setae absent; one anepisternal, one katepisternal setae, also anepisternum and katepisternum covered with short black setulae.

*Legs*. Yellow. Tarsus, mid- and hindcoxae darkened to pale brown.

*Wings*. Hyaline, with yellowish apex. Cell r_4+5_ narrowly opened. Apices of veins R_4+5_ and M almost in contact. Posteroapical extension of cell bcu 0.3 times as short as the width of cell at its middle. Halter yellow.

*Abdomen* evenly short and thin, black, setulose. All tergites laterally yellow, brown at middle, syntergite 1+2 and tergite 3 completely brown. Sternites yellow.

*Male genitalia*. Surstylus with anteroventral lobe curved proximally at right angle, bears two spines at its medial margin; and with short posteromedial lobe covered with setulae on its ventral side. Cerci tapered at top, separated by narrow slit, covered with long setulae.

Body length, 4.7–5.5 mm. Wing length, 3.5–4.5 mm.

**Female**: similar to male, except for genital structures.

Body length, 5.0–7.0 mm. Wing length, 3.5–4.8 mm.

#### Distribution.

Mongolia.

### Additional material examined

#### Timia canaliculata

Taxon classificationAnimaliaDipteraUlidiidae

Becker, 1906

Figure 5

##### Type material.

***Lectotype male*: China**: “syntypus, Бугасъ у Хами, на ЮВ от Тянь-Шаня, РобКозлов, 25.VIII.1895” [Bugaz near Hami, to the SE from Tien Shan, 25.VIII.1895, Roborowsky and Kozlov leg.] (ZISP).

***Paralectotypes*: China**: 3 males, 1 female, 25.VIII.1895, label as in lectotype; 1 male 28.VIII.1895, 1 female 20.VIII.1895, labels as in lectotype (ZISP); 4 males, 1 female, label as in lectotype (MNKB).

**Additional material: Mongolia**: 1 female, Mongolia, Ömnögovi Province, south coast of Buir Lake, 17.VII.970, I.M. Kerzhner leg.; 3 males, 1 female, Mongolia, Khovd Province, Elhon, 20 km SE Altai to Bodonchi, July 27, 1970, I.M. Kerzhner leg. (ZISP).

#### Timia dimidiata

Taxon classificationAnimaliaDipteraUlidiidae

Becker, 1906

Figure 6

##### Type material.

***Holotype male*: China**: “50812.” “Kaschgar, V.1903”, “dimidiata Beck.” [original Becker’s handwritten labels], “Typus” [red printed label] (MNKB).

**Additional material: Iran**:1 female, from Gurmuk to W and NW Sistan (Expedition to Persia, 1898), 5–20.IX.1898, Zarudny leg.; 2 males, 1 female, Senetang (Expedition to Persia, 1898), 13–17.V.1898, Zarudny leg. (ZISP); **Kazakhstan**: 1 male, Karakul-Sarah Tugay, Syr Darya, South Kazakhstan Province, 18.V.1898, Heyer leg.; 9 females, 3 males, SE Muyunkum, 110 km NW Jambyl, Zone in front of sands, on the *Alhagi* (camel thorn), 2.VII.1963, Sugonyaev leg. (ZISP); **Tajikistan**: 2 females, neighborhood of Gandzhin, NW Qurghonteppa, 12.V.1961, I.M. Kerzhner leg. (ZISP); **Turkmenistan**: 1 male, Karakul, 65 km. N of Ashgabat, 19.IV.1963, Ponomareva leg.; 3 males, lake Topyatan 15 km NNE Yaskhan, Uzboy, 18–19.V.1987, Vereshchagina leg.; 1 male, 12 km SE Tejen, 24.V.1964, Ponomareva leg.; 2 females, 20 km N Kizil Arvat, 3.VI.1952, Shteinberg leg.; 1 male, Merv, 15.VI.1930, V. Popov leg.; 1 male, 1 female, station Akhcha-Kuyma, 5.VII.1934, V. Popov leg.; 2 females, Karadegish, valley of. the Atrek river, 21.VIII.1932, Ushinsky leg.; 1 specimen without abdomen, Arman Saad-Kizil Arvat, Transcaspian region, 1896, Anger leg.; 1 female, neighborhood of Bugdali, SW Turkmenistan, 6.VII.973, Nartshuk leg.;1 male, 28 km SW Kumdag, Turkmenistan, saline, 5.VI.1973, Nartshuk leg. (ZISP); **Uzbekistan**:1 female, Buchara, Mer. occ., Yargak, pr. Chatyrtshy, 20.VI.1928, L. Zimin leg.; 11 males, 8 females, Kamak, Kattakurgan, near Samarkand, 29.VI.1929, L. Zimin leg.; 1 male, the same place, 10.VII.1929, L. Zimin leg.; 5 males, 4 females, the same place, 1.VII.1932, L. Zimin leg.; 1 male, 2 females, 100 km ENE Taxtako‘pir, Uzbekistan, O.G. Ovtshinnikova leg., 14.VI.1987; 5 males, 2 females, 32 km NNE Tashkömür, Uzbekistan,O.G. Ovtshinnikova leg., 12.VI.1987 (ZISP); **Armenia**:1 female, Arazdayan, 8.VI.1956, Zimina leg. (ZMUM).

#### Timia flaveola

Taxon classificationAnimaliaDipteraUlidiidae

Galinskaya, 2011

Figure 7

##### Type material.

***Holotype female*: Turkmenistan**: “Репетек, личинки в корнях Convolvulus. Лет – конец июня, собр. Каплин, 27.IV.1980” [Repetek, larvae on the roots of *Convolvulus* (Convolvulaceae), hatching in the late June, 27.IV.1980 Kaplin leg.] (ZISP). Paratypes: 5 females, label as in holotype; 1 female, **Turkmenistan**: “Репетек, Туркм. саксаульник близ усадьбы, Стальмакова, 21.VI.1947” [Repetek (*Haloxylon* near the estate), 21.VI.1947 Stal’makova leg.] (ZISP).

#### Timia gobica

Taxon classificationAnimaliaDipteraUlidiidae

Zaitzev, 1982

Figure 8

##### Type material.

***Holotype male*: Mongolia**: “Монголия, Южно-Гобийский аймак, 20 км СВ Булгана, песчаная пустыня с саксаулом, Кандыбина, 10.VII. 1971” [Ömnögovi Province, 20 km NE Bulgan, sand desert, on *Alhagi*, 10.VII.1971, Kandybina leg.] (ZISP).

**Additional material: Kazakhstan**: 4 males, 60 km. NW Dzhingilda, Kyzyl Kum, 24.V.1965, Zaitzev leg. (ZISP); **Mongolia**: 4 males, 8 females, Ömnögovi Province, station Bulgan, on *Alhagi*, 20 km NE Bulgan, 22.VI.1971, Kandybina leg.; 1 female, Südgobi Province, 100 km W v. Grenzposten Ovot Chuural, 1250 m; Nr. 835, 23.VI.1967, exp. Dr. Z. Kaszab; 2 males, 1 female, Ömnögovi Province, 20 km NE Bulgan (*Haloxylon ammodendron*), 2.VII.1971; 4 females, Ömnögovi Province, 20 km NE Bulgan (sandy desert with the *Alhagi*), 19.VII.1971, Kandybina leg.; 1 female, Govi-Altai Province, spring Haichi-Bulak, 60 km. SE Bugat, 19.VII.1970, Yemelyanov leg.; 2 males, Khovd Province, Elhon, 20 km SE Altai to Bodonchi, 27.VII.1970, Nartshuk leg.; 2 females, Ömnögovi Province, Dzemgin Gobi, 25 km. SSW Haylastyn-Khuduk, I.M. Kerzhner leg.(ZISP); **Uzbekistan**: 1 male, Khiva, Ravat, 29.VII.1927 L.Zimin leg. (ZISP).

#### Timia orientalis

Taxon classificationAnimaliaDipteraUlidiidae

Zaitzev, 1982

Figure 9

##### Type material.

***Holotype male*: Mongolia**: “Монголия, Южно-Гобийский аймак, Бордзон-Гоби, 80 км. ЮЮВ Номгона, Зайцев, 5–8.VIII.1967” [Ömnögovi Province, Bordzon Gobi, 80 km. SSE Nomgon, 5–8.VIII.1967, V.Zaitzev leg.] (ZISP).

***Paratypes***: 8 females, 3 males: **Mongolia**: “Монголия, Южно-Гобийский аймак, Бордзон-Гоби, 80 км. ЮЮВ Номгона, Зайцев, 5–8.VIII.1967” [Ömnögovi Province, Bordzon Gobi, 80 km. SSE Nomgon, 5–8.VIII.1967, Emelianov leg.] (ZISP). **China**: 3 females, “Баинхудук; с. Алашань, Козлов, 20.V.1909” [Bainhuduk, north Alashan Plateau, Gobi Desert, 20.V.1909, Kozlov leg.] (ZISP).

**Additional material: China**: 1 male, Etszin-gol, north Alashan Plateau, Gobi Desert, 18.VI.1909, Kozlov leg. (ZISP); **Mongolia**: 1 female, Ömnögovi Province, Bordzon Gobi, 80 km. SSE Nomgon (sands), 5–8.VIII.1967, Kerzhner leg.; 8 females, 3 males, Ömnögovi Province, Bordzon Gobi, 80 km. SSE Nomgon (sands), 5–8.VIII.1967, Yemelyanov leg. (ZISP).

#### Timia testacea

Taxon classificationAnimaliaDipteraUlidiidae

Portschinsky, 1891

Figure 10

##### Type material.

Lectotype female: “syntypus, Опр. Порчинский, 1892” [syntypus, 1892, Portshinsky leg.] (ZISP). Paralectotype: 1 female, label as in lectotype (ZISP). **China**: 1 male, “Kaschgar, V.1903, 50811, Typus, Typus von *Timia mellina* Becker” [Kashgar, V.1903, 50811, Type, Type of *Timia mellina* Becker] (MNKB).

**Additional material: Kazakhstan**: 1 male, Karauzyak, Kyzylorda Province, 15.VI.1916, N. Pulikovskaya leg.; 6 males, 1 female, Tash-Suat, boundary between Kyzylorda and Shymkent Province, 24.V.1896, Heyer leg.; 1 male, Kyzylorda, 17.VI.1926, Ruzaev leg.; 1 male, Kyzylorda, 27.VII.1926, Ruzaev leg.;1 female, Almaty Province, Sharyn River, Sortogoi, Lehr leg.(ZISP); 1 male, Karachingil, estuary of Syr Darya, 29.VI.1900, L.S. Berg leg.; 2 males, Kazaly, on *Tamarix*, 4.VII.1900, L.S. Berg leg. (ZMUM); **Tajikistan**: 2 males, 1 female, Isfara, IV–VI.1927 (ZISP); **Uzbekistan**: 2 males, station Fedchenko, 1926, L. Zimin leg.; 1 male, Khiva, VII.1929, Gerasimov leg.; 1 male, Pahtalyk-Kul, 15.VI.1925; 1 male, NO Yazyavan, Sary su River, Andijan Province, 22.V.1961, Sugonyaev leg.; 2 females, 1 male, near Tashkent (on *Glycyrrhiza glabra*), 1.VII.1963, Valieva leg. (ZISP); **Kyrgyzstan**: 1 female, Gulcha, N.N. Filippov leg. (ZMUM); **Turkmenistan**:1 female, Charshangu, 30 km from Kelif, Amu Darya, Petrischeva leg., 7.VI.934; 1 female, Ispas, 70 km NW Türkmenabat, Nartshuk leg. (ZISP); **China**: 1 female, Gobi desert, Taklamakan, Hauser leg., 1900, 54535, *Timia testacea* Portsch. V.Korneyev det. 1999; 1 female, 1 male, Gobi desert, Taklamakan, Hauser leg., 1900, 54535; 1 female, Gobi desert, Taklamakan, Hauser leg., 1900, 54535 *Timia testacea* Portsch. (MNKB).

#### Timia zaitzevi

Taxon classificationAnimaliaDipteraUlidiidae

Galinskaya, 2011

Figure 11

##### Type material.

Holotype male: **Kazakhstan**: “Ю.-З. Кызыл-Кумы, Ю. Казахст. обл., Лер, 20.V.1960” [SW Kyzyl Kum, 20.V.1960, Lehr leg.] (ZISP). Paratypes: 12 males, 3 females, label as in holotype (ZISP).

**Additional material: Kazakhstan**: 1 female, Turkestan station, Kazakhstan, Lukyanovich leg., 30.V.936; 3 females, SW. Kyzyl Kum, S. Kazakhstan Region, Lehr leg., 20.V.960; **China**: 1 female, sand desert near River Ili, 40 km from Lake Chianka, Poyarkov leg.; **Tajikistan**: 1 female, right bank of Amu Darya, near Aivadj, Shaartuz Region, Tajik SSR, 4.VI.1975, Volkovich leg. (ZISP).

## Supplementary Material

XML Treatment for Timia

XML Treatment for Timia lazebnayae

XML Treatment for Timia shatalkini

XML Treatment for Timia mongolica

XML Treatment for Timia canaliculata

XML Treatment for Timia dimidiata

XML Treatment for Timia flaveola

XML Treatment for Timia gobica

XML Treatment for Timia orientalis

XML Treatment for Timia testacea

XML Treatment for Timia zaitzevi

## References

[B1] Becker T (1906) *Timia* Wied. Wiener Entomologische Zeitung 25(2–4): 108–118. doi: 10.5962/bhl.part.5378

[B2] Chen X-L Kameneva EP (2009) A review of *Ulidia* Meigen (Diptera: Ulidiidae) from China. Zootaxa 2175: 42–50.

[B3] Galinskaya TV (2011) Review of the yellow-bodied species of the genus *Timia* (Diptera: Ulidiidae) with description of two new species. Zootaxa 2888: 1–22. 10.11646/zootaxa.3753.6.4 24869515

[B4] Galinskaya TV (2014) Two new species of the genus *Timia* (Diptera: Ulidiidae) with a key to species with microtrichose black scutellum. Zootaxa 3753(6): 573–584. 24869515 10.11646/zootaxa.3753.6.4

[B5] Hadley A (2007) CombineZM – Open source image processing software package for creating extended depth of field images. http://www.hadleyweb.pwp.blueyonder.co.uk

[B6] Hendel F (1908) Synopsis der bisher bekannten *Timia*-Arten. Zeitschrift für Hymenopterologie und Dipterologie 8: 1–12.

[B7] Hennig W (1940) Ulidiidae. In: Lindner E (Ed.) Die Fliegen der palaearktischen Region. E. Schweizerbart, Stuttgart, 1–34.

[B8] Kameneva EP (1996) A new species of the genus *Timia* (Diptera: Ulidiidae) from Tien-Shan mountains. Journal of the Ukrainian Entomological Society 2(3–4): 51–55.

[B9] Kameneva EP (2000) Picture-winged flies (Diptera, Ulidiidae) of Palearctics (fauna, morphology and systematics). PhD Thesis, I.I. Schmalhausen Institute of Zoology, National Academy of Sciences of Ukraine, Kyiv [In Ukrainian]

[B10] Kameneva EP (2010) *Timia berlandi* Sinai. Site on the taxonomy of the Picture-Winged Flies (Diptera: Ulidiidae), worldwide. https://sites.google.com/site/seioptera/pictures-references/my-pictures/temia-berlandi-sinai

[B11] Morgulis E Freidberg A (2014) The Ulidiini (Diptera: Tephritoidea: Ulidiidae) of Israel, with a key to the world species of *Ulidia* and description of five new species. Zootaxa 3780(2): 201–247. 24871834 10.11646/zootaxa.3780.2.1

[B12] White IM Norrbom AL Headrick DH Carroll LE (1999) Glossary. In: Aluja M Norrbom AL (Eds) Fruit flies (Tephritidae): Phylogeny and evolution of behavior. CRC Press, Boca Raton, 881–924. doi: 10.1201/9781420074468.sec8

[B13] Zaitzev VF (1982) Flies of the family Ulidiidae (Diptera) in the fauna of Mongolia. In: Kerzhner IM (Ed.) Insects of Mongolia, 8 Nauka, Leningrad, 422–453. [In Russian]

[B14] Zaitzev VF (1984a) Family Ulidiidae. In: Soós A Papp L (Eds) Catalogue of Palaearctic Diptera, Vol. 9, Micropezidae–Agromyzidae Akadémiai Kiádo and Elsevier Science Publishers, Budapest and Amsterdam, 59–66.

[B15] Zaitzev VF (1984b) A new species of the family Ulidiidae (Diptera) from Middle Asia. Journal of Zoology 62(4): 628–630.

